# Bilateral Spontaneous Thalamic Hemorrhage: A Case Report

**DOI:** 10.7759/cureus.67258

**Published:** 2024-08-19

**Authors:** Zakariya A Al-Shahwani, Ghaith M Taha, Raghad N Saoodi, Mohanad Faisal

**Affiliations:** 1 Emergency Medicine, Al Karama Teaching Hospital, Baghdad, IRQ; 2 Neurology, Al Kadhmiya Teaching Hospital, Baghdad, IRQ; 3 Emergency Medicine, Al-Shaheed Mohammed Baqir Alhakeem General Hospital, Baghdad, IRQ; 4 Internal Medicine, Hamad Medical Corporation, Doha, QAT

**Keywords:** hypertensive intracerebral haemorrhage, thalamic hemorrhage, spontaneous cerebral hemorrhage, bilateral thalamic, intracranial hemorrhage (ich)

## Abstract

Bilateral spontaneous thalamic hemorrhage is a rare but serious condition often linked to hypertension. We report a 67-year-old male presenting with a sudden severe headache, decreased consciousness, and right-sided neurological deficits. Both CT and MRI confirmed bilateral thalamic hemorrhage. The patient was managed with supportive care and blood pressure control. This case underscores the importance of early recognition and adds to the limited literature on this condition.

## Introduction

Intracranial hemorrhage (ICH) is a serious and common neurological emergency that carries significant morbidity and mortality. It often presents with an acute onset of severe symptoms, posing significant challenges in management. It’s usually solitary, but it can present at any site in the brain with any number. The thalamus, a vital brain structure involved in sensory and motor signal relay, when affected bilaterally, can lead to profound neurological deficits and complications [1]. Hypertension is the most recognized risk factor for such hemorrhages [2], though the exact mechanisms leading to bilateral involvement remain a topic of ongoing research and debate. This case report of a 67-year-old male who presented with bilateral spontaneous thalamic hemorrhage underlines the critical importance of early detection through the utilization of brain imaging with the management and follow-up of this rare clinical entity.

## Case presentation

A 67-year-old male presented with a sudden-onset, severe generalized headache lasting 30 minutes, followed by a decreased level of consciousness. The patient collapsed, exhibiting frothy secretions from his mouth and urine incontinence, though no abnormal body movements were observed. His medical history was significant for ischemic heart disease, for which he was non-compliant with treatment. The patient was a smoker and did not consume alcohol.

On examination, his Glasgow Coma Scale (GCS) score was 10, with a blood pressure of 170/80 mmHg and a pulse rate of 60 beats per minute. Cardiovascular, respiratory, and abdominal examinations were unremarkable. Neurological examination revealed right upper motor facial palsy and right-sided body weakness, accompanied by hyperreflexia and bilateral upgoing plantar reflexes.

**Figure 1:**
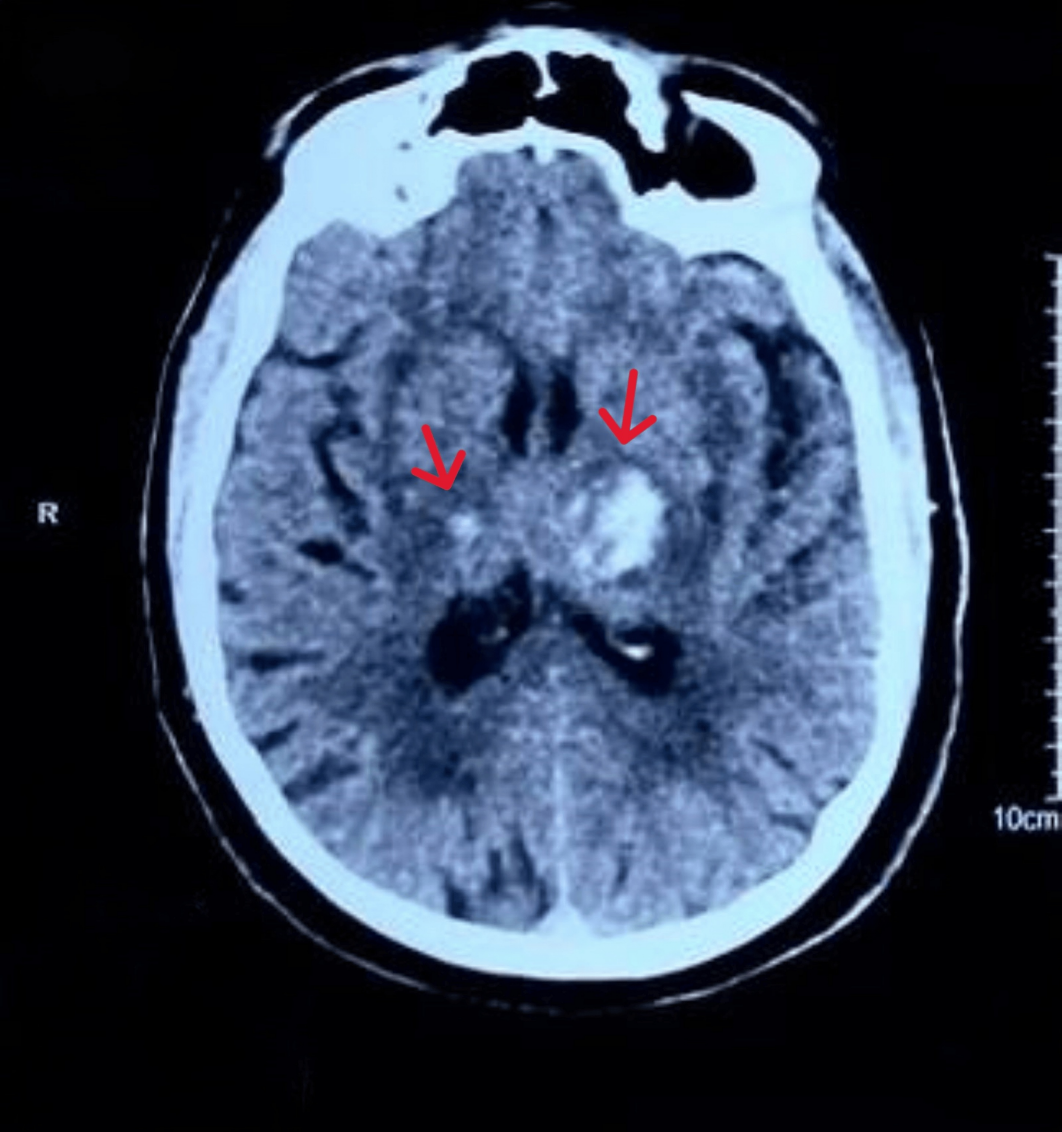
A CT scan of the brain (axial view) shows the presence of bilateral hyperdensities at the basal ganglia area (marked by red arrows).

**Figure 2:**
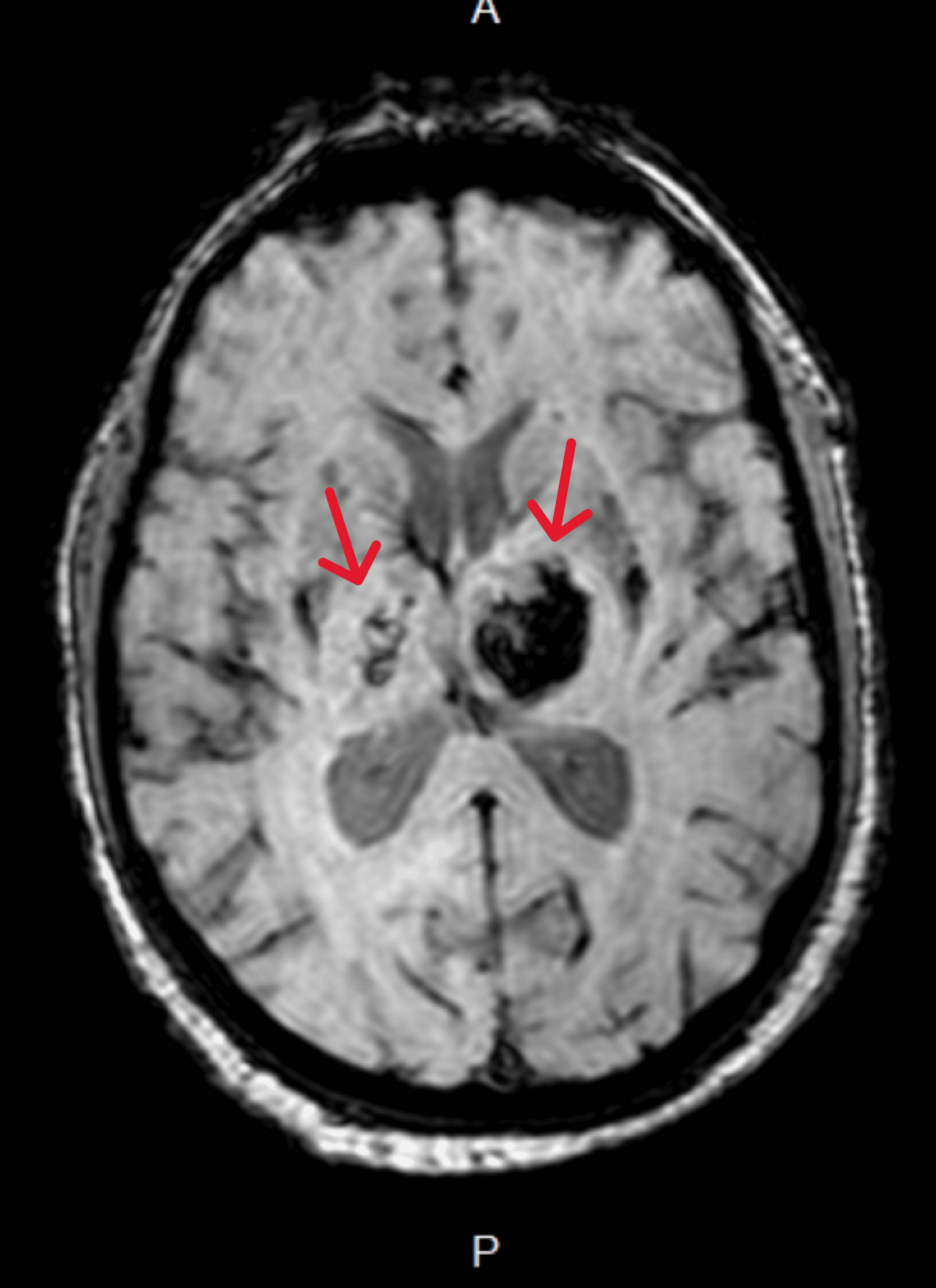
An MRI of the brain, susceptibility-weighted imaging shows bilateral thalamic hypointensities (marked by red arrows) going with bilateral thalamic hemorrhage.

Laboratory investigations showed normal complete blood counts and coagulation profiles. Liver and renal function tests were within normal limits. A CT scan of the brain (Figure 1) revealed bilateral ICH in the basal ganglia area. An MRI of the brain (Figure 2) confirmed the presence of bilateral thalamic hemorrhage, with the largest hemorrhage on the left side measuring 33.3 by 23.4 mm. The patient was managed with supportive care and blood pressure control and was successfully discharged home with follow-up by physical and occupational therapists.

## Discussion

Non-traumatic ICH typically occurs in solitary and has a high rate of mortality and morbidity. One of the most well-known risk factors is hypertension. Other causes of ICH are trauma and vasculopathy, including arteriovenous malformation, cavernoma, and aneurysm [3]. The incidence of multiple simultaneous ICH varies from 1% to 4.7% in cases of spontaneous ICH [4]. Bilateral hemorrhages mostly happen due to hypertension and can be explained by different theories.

According to the first theory, perforator vessels are prone to rupture, causing a spike in blood pressure throughout the body and subsequent bleeding on the other side. Another hypothesis states that the perforator vessels are usually weak and that, in rare instances, rupture happens simultaneously on both sides, probably as a result of an increase in blood pressure. We think that the first theory might explain the situation in our case, as there is a size difference between the hematomas of each size in addition to the slight difference in density [5].

Bilateral thalamic hemorrhages are rarely reported in the literature [6]. Important complications of such presentation include an altered level of consciousness in the acute phase, as shown in our case, in addition to the late manifestation of thalamic dementia [7]. Thalamic involvement in ICH carries a poor prognosis among ICH patients [8]. Management focuses on blood pressure control and supportive care. Surgical treatment is often warranted for life-saving situations when the GCS drops below 12 or the hematoma volume is above 30 ml [9]. Our patient was managed conservatively as the surgical risk was high, and he showed a good response to conservative management.

## Conclusions

Bilateral spontaneous thalamic hemorrhage is a rare but serious condition that neurologists and emergency doctors need to be aware of. This case highlights the importance of early recognition and management, including blood pressure control and supportive care, which facilitated a positive outcome. This report adds to the limited literature and underscores the need for awareness and prompt intervention.
